# Editorial: Socioeconomic status and health in childhood, adolescence and young adulthood

**DOI:** 10.3389/fpubh.2022.1042331

**Published:** 2022-10-26

**Authors:** Heide Busse, Florence Samkange-Zeeb, Graham Moore

**Affiliations:** ^1^Leibniz Institute for Prevention Research and Epidemiology – BIPS, Bremen, Germany; ^2^School of Social Sciences, Cardiff University, Cardiff, United Kingdom

**Keywords:** equality, inequity, intervention, childhood, adolescence

Socioeconomic status (SES) and health are connected across the lifecycle, from childhood and adolescence, through to older age. From decades of research it is known that the social conditions we are born and grow up in influence our health, with those in a lower social position often having worse health and wellbeing, and greater barriers to leading a healthy and fulfilling life (1–3).

SES impacts health in multiple ways, at multiple levels—cutting across individual, social, community and structural domains. Health inequalities are driven by political and economic forces which shape the unequal distribution of power and status and material affluence in societies (4). Hence, structural forces also need to be considered when addressing the root causes of health inequalities. The establishment of healthy and sustainable places, settings and communities, as well as initiatives early in the life-course, can help to help interrupt cycles of disadvantage and mitigate negative effects, before socio-economic patterning widens even further in adulthood (5, 6).

The COVID-19 pandemic helped bring the social gradient in health once again to the fore. While the pandemic compromised the health, social and material wellbeing of young people in general, there is some evidence that those from lower SES backgrounds were hit the hardest (7, 8). Disproportionately negative effects of the COVID-19 pandemic are well illustrated in the contribution by Geweniger et al. Findings of the cross-sectional online study, involving 1,619 caregivers of children and adolescents aged 1–18 years in Germany, showed that caregiver-reported mental health problems in children were more likely to affect children of low SES, with complex chronic diseases and with caregivers screening positive for depression. Whilst the study highlights existing inequalities in mental health outcomes by SES, it also accentuates how the pandemic might have widened existing inequalities and mental health problems.

The contribution by  Schoon and Henseke also highlights the unequal impact of the COVID-19 pandemic on adolescents' and young adults' health. The authors analyzed data from the Youth Economic Activity and Health survey, a nationally representative longitudinal sample of 16–25 year-olds in the United Kingdom. Using a stress process model, the study investigated the role of different psychosocial resource factors in mitigating the vulnerability to mental distress among disadvantaged young people and possible mediating pathways. Analyses revealed sequential mediating processes, where SES influences were found to be partially mediated *via* financial strain and psychosocial resource factors. Psychosocial resource factors showed independent effects supporting mental health during socio-economic adversity, and social support was found to play a significant role in increasing self-efficacy and optimism. The authors recommend the consideration of multiple resource factors instead of single aspects to gain a better understanding of the processes linking SES to young people's mental health.

As well as mental health outcomes, overweight and obesity are current topics of concern regarding child and adolescent health. In their contribution, Doi et al. investigated whether “Adverse Childhood Experiences” (ACEs) are associated with obesity in Japanese school children aged 9–14 years. The authors analyzed cross-sectional data from the Adachi Child Health Impact of Living Difficulty (A-CHILD) study, and found that the number of experiences categorized by the authors as ACEs was not associated with overweight or obesity among adolescents. However, single parenthood and low household income were found to show an independent association with obesity. The authors call for the prevention of exposure to ACEs to be clearly addressed in child health policies.

In the final contribution to this Research Topic, Ettinger et al. present the conceptual framework and study protocol of the Tracking Health, Relationships, Identity, EnVironment, and Equity (THRIVE) Study. The authors insight into a study that promises to be of great value to research on social determinants of health (of children) as it encompasses many facets imperative for evidence-based (public health) research. The study is part of the “The Pittsburgh Study”, whose aim is to understand and promote child and youth thriving, as well as build health equity and strengthen communities through the application of community-partnered participatory research approaches. Children from 0 to 18 years will be followed-up in six cohorts, and data from a variety of sources including electronic health records, school records, as well as health and human services data will be combined. A further characteristic of the study is that principles of racial justice, equity and inclusion will be considered. The results of this study will surely be avidly awaited by many researchers, practitioners as well as policy-makers.

How do interventions impact on inequalities? Are existing (digital) interventions equally effective for different socioeconomic groups? These were some of the key questions that the Research Topic initially set out to answer. However, none of the contributing papers focussed on investigating differential effects of intervention studies by socioeconomic subgroups, and perhaps unsurprisingly given the period in history when the papers were submitted, there was a strong emphasis on impacts of the COVID-19 pandemic. Understanding inequalities in recovery from the pandemic will remain a priority for some time.

Despite strong evidence of the social gradient in health and the adoption of a multitude of different intervention approaches across varying international settings, up to now little attention has been paid to assess equity impacts of public health interventions. Whilst we acknowledge the contribution of cross-sectional and longitudinal studies toward understanding associations between socioeconomic status and health, we believe it is now high time to go further and to investigate effects of interventions on socioeconomic status and health in children, adolescents and young adults.

We thus strongly encourage researchers to (i) theorize the potential for interventions to widen, or reduce, inequalities and build these considerations into intervention development, (ii) investigate how interventions work within different subgroups of the population, (iii) examine mechanisms through which inequalities are perpetuated and sustained, and, (iv) aim to advance the understanding on how interventions impact on inequalities and what works best to narrow inequalities in health. Knowledge on what works is insufficient if we do not know **who** it works for and **whether** existing inequities in health are potentially sustained or even widened.

## Author contributions

HB, FS-Z, and GM contributed to the conception of the Research Topic. HB wrote the first draft of the editorial. FS-Z and GM critically reviewed and edited the editorial. All authors contributed to the editorial, revised, read, and approved the submitted version.

## Conflict of interest

The authors declare that the research was conducted in the absence of any commercial or financial relationships that could be construed as a potential conflict of interest.

## Publisher's note

All claims expressed in this article are solely those of the authors and do not necessarily represent those of their affiliated organizations, or those of the publisher, the editors and the reviewers. Any product that may be evaluated in this article, or claim that may be made by its manufacturer, is not guaranteed or endorsed by the publisher.
